# Less Reactive Thiol Ligands: Key towards Highly Mucoadhesive Drug Delivery Systems

**DOI:** 10.3390/polym12061259

**Published:** 2020-05-30

**Authors:** Iram Shahzadi, Andrea Fürst, Zeynep Burcu Akkus-Dagdeviren, Shumaila Arshad, Markus Kurpiers, Barbara Matuszczak, Andreas Bernkop-Schnürch

**Affiliations:** 1 Center for Chemistry and Biomedicine, Department of Pharmaceutical Technology, Institute of Pharmacy, University of Innsbruck, Innrain 80-82, A-6020 Innsbruck, Austria; Iram.Shahzadi@student.uibk.ac.at (I.S.); Andrea.Fuerst@uibk.ac.at (A.F.); Zeynep.Akkus@uibk.ac.at (Z.B.A.-D.); Shumaila.arshad@pharm.uol.edu.pk (S.A.); m.kurpiers@thiomatrix.com (M.K.); 2 Faculty of Pharmacy, The University of Lahore, 54000 Lahore, Pakistan; 3 Thiomatrix Forschungs- und Beratungs GmbH, Trientlgasse 65, A-6020 Innsbruck, Austria; 4 Center for Chemistry and Biomedicine, Department of Pharmaceutical Chemistry, Institute of Pharmacy, University of Innsbruck, Innrain 80-82, A-6020 Innsbruck, Austria; barbara.matuszczak@uibk.ac.at

**Keywords:** less reactive thiomers, mucoadhesion, mucus diffusion, polyacrylic acid, s-protected thiomers, thiolation

## Abstract

As less reactive s-protected thiomers can likely interpenetrate the mucus gel layer to a higher extent before getting immobilized via disulfide bond formation with mucins, it was the aim of this study to develop a novel type of s-protected thiomer based on the less reactive substructure cysteine-*N*-acetyl cysteine (Cys-NAC) in order to obtain improved mucoadhesive properties. For this purpose, two types of s-protected thiomers, polyacrylic acid-cysteine-mercaptonicotinic acid (PAA-Cys-MNA) and polyacrylic acid-cysteine-*N*-acetyl cysteine (PAA-Cys-NAC), were synthesized and characterized by Fourier-transform infrared spectroscopy (FT-IR) and the quantification of attached disulfide ligands. The viscosity of both products was measured in the presence of NAC and mucus. Both thiomers were also evaluated regarding swelling behavior, tensile studies and retention time on the porcine intestinal mucosa. The FT-IR spectra confirmed the successful attachment of Cys-MNA and Cys-NAC ligands to PAA. The number of attached sulfhydryl groups was in the range of 660–683 µmol/g. The viscosity of both s-protected thiomers increased due to the addition of increasing amounts of NAC. The viscosity of the mucus increased in the presence of 1% PAA-Cys-MNA and PAA-Cys-NAC 5.6- and 10.9-fold, respectively, in comparison to only 1% PAA. Both s-protected thiomers showed higher water uptake than unmodified PAA. The maximum detachment force (MDF) and the total work of adhesion (TWA) increased in the case of PAA-Cys-MNA up to 1.4- and 1.6-fold and up to 2.4- and 2.8-fold in the case of PAA-Cys-NAC. The retention of PAA, PAA-Cys-MNA, and PAA-Cys-NAC on porcine intestinal mucosa was 25%, 49%, and 76% within 3 h, respectively. The results of this study provide evidence that less reactive s-protected thiomers exhibit higher mucoadhesive properties than highly reactive s-protected thiomers.

## 1. Introduction

Since mucoadhesive polymers entered the pharmaceutical arena in the early 1980s [1,2], extensive research has been done to utilize these excipients for various types of mucosal drug delivery systems [3]. Among mucoadhesive polymers [4], thiolated polymers designated as thiomers exhibit the highest mucoadhesive properties. Thiomers are believed to interact with the cysteine-rich subdomains of mucus glycoproteins, forming disulfide bonds between the mucoadhesive polymer and the mucus layer [5,6]. They offer also a more cohesive polymeric matrix due to the disulfide bond formation within their polymeric network providing a slower and more controlled drug release [7,8]. Thiomers are, however, not sufficiently stable, as their thiol groups are sensitive towards oxidation at pH > 5, limiting their potential. To overcome this problem, s-protected thiomers were generated also termed as “preactivated thiomers” [9]. For s-protection, mercaptopyridine analogues such as 2-mercaptonicotinic acid (MNA) or 2-mercaptonicotinamide are commonly used as these groups do not just provide protection against oxidation but they also raise the reactivity of the thiol groups for disulfide exchange reactions with mucus [9,10]. There are numerous studies available describing the superiority of preactivated thiomers over thiomers without s-protection regarding mucoadhesive properties [11,12,13]. This high reactivity, however, can cause the immobilization of such thiomers already on the surface of the mucus layer, hindering their interpenetration into deeper mucus regions where they could be anchored more tightly. Moreover, in the case of just being attached to the mucus surface, they are prone to much faster elimination due to the rapid mucus turnover process [14]. In addition, without deeper interpenetration, thiomers will not be able to interact with membrane-bound substructures of epithelial cells providing additional effects such as an opening of tight junctions [15]. Even in the case of in-situ gelling properties, too high reactivity of preactivated thiomers might be disadvantageous, as a gelation that is too fast can limit a proper distribution of the polymer over the target tissue [16]. According to these considerations, moderate reactive s-protected thiomers might provide higher mucoadhesive properties. For this purpose, a novel comparatively less reactive ligand cysteine-*N*-acetyl cysteine (Cys-NAC) was synthesized and attached to a polyacrylic acid (PAA) backbone. For comparison reasons, a highly reactive polyacrylic acid-cysteine-mercaptonicotinic acid (PAA-Cys-MNA) conjugate was prepared as its mucoadhesive properties are already reported in the literature [17]. Both s-protected thiomers PAA-Cys-NAC and PAA-Cys-MNA were evaluated via rheological investigations, tensile studies, and mucoadhesion studies on the freshly excised porcine intestinal mucosa. Furthermore, the diffusion behavior of both thiomers in mucus was determined.

## 2. Materials and Methods

### 2.1. Materials

Poly (acrylic acid) (PAA) 450 kDa, dichloromethane (DCM) (≤0.001% residual water), dimethylformamide (DMF) (≤0.005% residual water), dimethyl sulfoxide (DMSO) (≤0.20% residual water), 2-mercaptonicotinicacid (MNA), l-cysteine ≥ 98%, Dowex^®^ 1 × 4 chloride form (100–200 mesh), 5,5′-dithiobis (2-nitrobenzoic acid) (Ellman’s reagent; DTNB), sodium borohydride ≥ 98%, N-acetyl-l-cysteine ≥ 99%, Triethylamine ≥ 99%, and all other salts and solvents were purchased from Sigma Aldrich, Vienna, Austria. Acetonitrile (≤0.30% residual water) was obtained from Donauchemie GmbH, Vienna, Austria. The porcine small intestine was kindly donated by a local slaughter house.

### 2.2. Synthesis of S-Protected Thiol Ligands

#### 2.2.1. Synthesis of Cys-MNA

The ligand (Cys–MNA) was synthesized in a two-step reaction. 2-Mercaptonicotinic acid (MNA) was first oxidized to the corresponding MNA-dimer with hydrogen peroxide according to a method described previously [18]. The obtained MNA-dimer was subsequently modified by a disulfide exchange reaction with l-cysteine to Cys–MNA. Briefly, 2 g of MNA was dispersed in 80 mL of demineralized water by ultrasonication for 10 min. Afterward, the pH was adjusted to pH 8 with 5 M NaOH obtaining a clear, yellow solution. In order to oxidize MNA, 2.5 mL of hydrogen peroxide (30% *v*/*v*) was added and stirred for 10 min until the solution became colorless. The pH was maintained during the reaction at pH 8–9. Following that, a solution containing 1 g of l-cysteine in 40 mL of demineralized water was adjusted to pH 8 and added drop-wise to the reaction mixture within 1 h. The pH was maintained during the reaction at pH 8.5–9.5 and a slightly yellow clear solution was obtained. Thin-layer chromatography (TLC) was used to verify that Cys–MNA was successfully formed. The stationary phase was silica gel with a fluorescent indicator coated on aluminum foils and a mixture of butanol:acetic acid:water (4:1:1) was used as the mobile phase. The plates were analyzed under black light (UV_254_) and subsequently sprayed with a ninhydrin solution (0.3% *m*/*v* in ethanol).

#### 2.2.2. Purification of Cys-MNA

In order to eliminate unbound l-cysteine from the reaction mixture, two purification steps were performed. The pH of the slightly yellow solution was decreased to pH 7.5 to precipitate l-cystine since it is poorly soluble in aqueous media of pH 2–8 [10]. The precipitation was removed by filtration and the filtrate was further purified with an ion-exchange column. At pH 7.5, l-cysteine is uncharged, whereas MNA, MNA-dimer and Cys–MNA are mainly negatively charged. Therefore, the column was packed with 30 g of anionic exchange resin (Dowex 1 × 4 chloride form, 100–200 mesh), loaded with 250 mL of a 2 M NaCl solution, and washed with 250 mL of demineralized water. Afterward, the filtrate was added to the column and was washed two times with 250 mL of demineralized water in order to remove l-cysteine. The bound compounds were released by adding three times 250 mL of a 2 M NaCl solution and the collected eluent was reduced to 200 mL with a rotary evaporator. The solution was then frozen at −80 °C and lyophilized using a rotary vacuum evaporator (G3B-Heidolph Hei-VAP Value with Vacuubrand pump, Vaccubrand GmbH, Werthheim, Germany). 

#### 2.2.3. Synthesis of Cys-NAC

l-Cysteine (605.8 mg) was dissolved in 20 mL of deionized water and its pH was adjusted to 8.2. NAC (815.975 mg) was dissolved in 20 mL of deionized water and its pH was adjusted to 8.2. Afterward, both solutions were mixed. A total of 0.567 mL of hydrogen peroxide (30% *v*/*v*) was then added to the mixture while maintaining pH at 8.5 to 9.5 with 5 M NaOH. The solution was continuously stirred for 2 h at room temperature. Thin-layer chromatography (TLC) was used to verify the formation of Cys-NAC in the same way as described for Cys-MNA. The pH of the solution was decreased to pH 7.5 to precipitate l-cysteine and cysteine dimer. The precipitates were filtered out and the obtained filtrate was then freeze-dried at −80 °C.

#### 2.2.4. Purification of Cys-NAC

In order to separate the remaining impurities from the product, the lyophilized product was dissolved in a mixture of acetonitrile and water (3:2 *v*/*v*). Precipitates were separated by filtration and Cys-NAC was obtained from the filtrate by evaporating the solvent under vacuum. The structure of the Cys-NAC disulfide was further characterized by ^1^H NMR spectroscopy.

#### 2.2.5. Liquid Chromatography–Mass Spectrometry (LC–MS)

The molecular mass of Cys-NAC was determined using LC–MS according to our previously used method with minor modification [19]. Mass spectrometry detection was carried out using a Chromaster 5610 MS detector (Hitachi) controlled by a computer running the MSD system manager software (version 2.1). The analysis conditions were as follows: ionization potential (V): 2500; counter gas flow (L/min): 0.6; Atmospheric pressure Ion Filter (AIF) temperature (°C): 120; ion source temperature (°C): 70; Aperture 1 (AP1) temperature (°C): 120; AIF introducing energy (eV): 5.0; AIF excreting voltage (V): 5.0; AP1 voltage (V): 80.0 and Aperture 2 (AP2) voltage (V): 30.0. The sample solution was prepared by dissolving the Cys-NAC in methanol: water (9:1 *v*/*v*) with 0.1% (*v*/*v*) formic acid at the concentration of 1 µg/mL. Mass spectral studies were performed in positive electrospray ionization (ESI) mode in the mass to charge ratio (*m*/*z*) range of 200–300 to determine molecular mass. In order to obtain clear mass spectra without any background noise, samples were directly infused into the mass spectrometer using a syringe pump at a flow rate of 2 µL/min.

### 2.3. Synthesis of Polyacrylic Acid Anhydride

PAA anhydride was prepared by adapting a method previously described [20]. For this, 1 g PAA powder was refluxed in acetic anhydride (20 mL) under nitrogen atmosphere at 150 °C. After 6 h, acetic anhydride was evaporated under vacuum. PAA anhydride was obtained as off-white powder and characterized by FT-IR.

### 2.4. Synthesis of s-Protected Thiolated PAA Conjugates

#### 2.4.1. Synthesis of PAA-Cys-MNA

PAA anhydride (142 mg) was dissolved in 10 mL of DMF by stirring at 80 °C. Ligand Cys-MNA (822 mg) was dissolved in 10 mL of DMSO. The precipitates were separated by filtration. In the clear filtrate, triethylamine (278 µL) was added and stirred for a further 15 min. After this, the ligand solution was added to anhydride solution and left for stirring for 24 h at 80 °C. The usage of triethylamine was required to prevent the protonation of amine groups. The product was purified by dialysis against water at 10 °C in dark using Nadir^®^ cellulose membranes, Carl Roth GmbH & Co. KG, Karlsruhe, Germany (molecular weight cut-off (MWCO) 3.5 kDa) for 3 days. The dialysis solution was changed every 8 h. After 3 days, the dialyzed product was freeze-dried at −80 °C. Finally, the product was characterized by FT-IR.

#### 2.4.2. Synthesis of PAA-Cys-NAC

PAA anhydride (142 mg) was dissolved in 10 mL of DMF by stirring at 80 °C. Cys-NAC (282 mg) was also dissolved in 10 mL of DMF and triethylamine (278 µL) was added. After 15 min, the ligand solution was added to anhydride solution and left for stirring for 24 h at 80 °C. The product was purified by dialysis against water at 10 °C in dark using Nadir^®^ cellulose membranes, Carl Roth GmbH & Co. KG, Karlsruhe, Germany (molecular weight cut-off (MWCO) 3.5 kDa) for 3 days. The dialysis solution was changed every 8 h. After 3 days, the dialyzed product was freeze-dried at −80 °C. Finally, the product was characterized by FT-IR.

### 2.5. FT-IR and ^1^H-NMR Spectroscopy

All FT-IR spectra were recorded on a Bruker ALPHA FT-IR apparatus equipped with a Platinum ATR (attenuated total reflection) module. NMR spectra were recorded on a “Mars” 400 MHz Avance 4 Neo spectrometer, from Bruker Corporation (Billerica, MA, USA). D_2_O was used as solvent and TMS as an internal standard.

### 2.6. Chemical Characterization of Thiolated PAA Conjugates

The thiolated products were analyzed for functional groups using Ellman’s test and disulfide bond test [21]. For Ellman’s test, 0.5–1 mg of each s-protected thiolated PAA conjugate was dissolved in 250 μL of Ellman’s buffer and 500 μL of Ellman’s reagent (5,5′-dithiobis(2-nitrobenzoic acid) 3 mg/10 mL of Ellman’s buffer) were added to each sample and were kept for incubation in the dark for 2 h. Thereafter, 100 μL aliquots were transferred to a 96-well plate, and absorption was measured at 450 nm using a microplate reader (Tecan infinite^®^ M200 spectrophotometer, Tecan Austria GmbH, Grödig, Austria). The amount of free thiol groups was then calculated using an already established calibration curve of l-cysteine under the same conditions. The degree of s-protection was determined by quantifying the amount of disulfide bonds. For this purpose, the above described procedure was followed after the reduction of disulfide bonds with sodium borohydride (NaBH_4_). The experiments were performed in triplicate.

### 2.7. Cytotoxicity Studies on Caco-2 Cell Line

The cytotoxic potential of unmodified and s-protected thiolated PAA conjugates was determined using the resazurin assay according to a previously described method [19]. Briefly, Caco-2 cells were seeded in a 24-well plate at a density of 25,000 cells per well in minimum essential medium (MEM) supplemented with penicillin/streptomycin solution (100 units/0.1 mg/L) and 10% (*v*/*v*) fetal calf serum (FCS). Cells were incubated for 14 days at 37 °C under 5% CO_2_ and a 95% relative humidity environment. During the incubation period, the medium was replaced every 48 h. Test solutions were prepared in the concentrations of 0.5%, 0.25% and 0.1% *m*/*v* in 25 mM HEPES buffered saline (HBS) pH 7.4. For the experiment, cells were washed twice with preheated HBS at 37 °C. Test solutions, positive control (HBS), and negative control (1% *v*/*v* Triton^TM^ X-100) were added in triplicate to the cell culture plate in the volume of 0.5 mL/well and incubated at 37 °C in a 5% CO_2_ and 95% relative humidity environment for 4 h. After incubation, test solutions were removed and cells were washed twice with preheated HBS pH 7.4. An aliquot of 0.25 mL of 2.2 mM resazurin solution was added to each well and the cells were incubated again under the same conditions for 3 h. Afterward, the fluorescence of the supernatant from each well was measured at 540 nm excitation wavelength and 590 nm emission wavelength.

Cell viability was calculated by the equation:

(1) $$\mathrm{Cell}\;\mathrm{viability}\;\left(\%\right)=\left(\frac{\;\mathrm{Experimental}\;\mathrm{value}-\mathrm{Negative}\;\mathrm{control}\;\mathrm{value}}{\mathrm{Positive}\;\mathrm{control}\;\mathrm{value}-\mathrm{Negative}\;\mathrm{control}\;\mathrm{value}}\right)\times 100$$

### 2.8. In-vitro Rheological Investigations

Rheological investigations were performed to determine the viscoelasticity of s-protected thiolated PAA conjugates as described previously by our research group [22]. Experiments were performed using Haake Mars Rheometer (Thermo Scientific™ HAAKE™ MARS™ rheometer, Thermo Fisher Scientific, Karlsruhe, Germany; Rotor C35/1°, D = 35 mm) with shear stress at a range of 0.5–500 Pa. In brief, 1% (*m*/*v*) solutions of each polymer in 50 mM phosphate buffer pH 6.8 were incubated with increasing concentrations of NAC 0.1, 0.5 and 1.0% (*m*/*v*) at 37 °C for 30 min. Unmodified PAA with the same concentration of NAC served as a control. The results were expressed as dynamic viscosity (*η*) increase with increasing NAC concentration for each sample. Each experiment was performed in triplicate.

Moreover, porcine intestinal mucus was collected and purified as described by Iglesias et al. [23] and also used for viscoelastic characteristics. In short, mucus was mixed with 1% *m*/*v* solution of each s-protected thiomer in a 2:1 ratio. The mixtures were incubated at 37 °C and viscosity was measured at pre-determined time points. Mucus mixed with 50 mM phosphate buffer pH 6.8 in the same ratio served as a control. The results were expressed as dynamic viscosity (*η*) increase with time. Each experiment was performed in triplicate.

### 2.9. Swelling Behavior

The swelling behavior of unmodified and both s-protected thiomers was determined by a method as previously described [24]. For this purpose, 5 mm flat-faced test disks of unmodified PAA and s-protected thiolated PAA conjugates were prepared. For the preparation of disks, 30 mg of each product was compressed by a single punch eccentric press at 11 KN (Paul-Otto Weber, Remshalden-Grünbach, Germany) with constant compaction pressure. The test disks were fixed on the clips and weighed. Afterward, the clips were immersed in 50 mM phosphate buffer pH 6.8. At predetermined time points, the clips with swollen test disks were removed from the buffer and weighed after gently removing excess water from the surface. The amount of water uptake at each time point was determined by the following equation:

(2) $$\mathrm{Water}\;\mathrm{uptake}\;\left(\mathrm{mg}\right)=W_{t}-W_{0}$$

where W_t_ is the disk weight at the specific time point and W_0_ is the initial disk weight.

### 2.10. In-vitro Mucoadhesion Studies 

#### 2.10.1. Tensile Studies

Tensile studies were performed using porcine intestinal mucosa to evaluate the adhesive force of unmodified and s-protected thiolated PAA conjugates by a texture analyzer (TA.XTplus Texture analyzer; Stable Micro Systems, Godalming, UK) following a previously described method with minor modifications [17]. In this method, the total work of adhesion (TWA) and the maximum detachment force (MDF) were investigated. For this purpose, test disks of unmodified PAA and s-protected thiolated PAA conjugates were prepared as described above for determining the swelling behavior. The intestine was cut into small pieces of approximately 16 cm^2^ diameters and fixed between two acrylic glass disks of the texture analyzer. The test disks were fixed on the lower side of the 10 mm cylindrical probe adapter with adhesive tape. During the experiment, the probe adapter with a speed of 0.5 mm/s was lowered to the mucosa, and a force of 0.5 N was applied for a contact time of 15 min. Moreover, test speed was kept constant 0.1 mm/s. Subsequently, the probe adapter was moved up with a withdrawal speed of 0.1 mm/s until the adhesive bond between mucosa and test disk failed. Data analysis was carried out using Exponent software.

#### 2.10.2. Mucoadhesion Using Flow-Through Method

##### Fluorescent Labeling of Unmodified and s-Protected Thiolated PAA

Unmodified and s-protected thiolated PAA conjugates were labeled with fluoresceinamine according to a method described previously by Imam et al. [25]. In brief, 1 g of each product was hydrated in 100 mL of demineralized water. Then, carbodiimide reagent (EDAC) was added in a final concentration of 40 mM under constant stirring for 20 min to activate the carboxylic acid moieties on each s-protected thiolated PAA-conjugate. Afterward, 20 mg of fluoresceinamine dissolved in 2 mL of 1 M NaOH was added to each solution, and the reactions were allowed to proceed for 3 h at room temperature at pH 5. The reaction mixtures were then dialyzed first against 0.2 mM HCl to remove unbound fluoresceinamine, then against the same medium with 1% *m*/*v* NaCl two times and finally against demineralized water. After dialysis, the solutions were freeze-dried (−80 °C, 0.1 mbar) and stored at 4 °C until further use. 

##### Mucoadhesion Study

To evaluate the mucoadhesive properties of s-protected thiolated PAA conjugates on intestinal mucosal membranes, in vitro mucoadhesion studies were performed as previously described by our research group [26]. Freshly excised porcine intestinal mucosa was collected from a local slaughterhouse and cut into pieces of 3 × 5 cm^2^. Each piece of mucosa was mounted on a 50 mL half-cut falcon tube adjusted at an angle of 45° in an incubator maintained at 100% relative humidity and 37 °C. In the following, 20 mg of fluoresceinamine-labeled samples were separately placed on each mucosa and incubated for 10 min to fully adsorb on the mucosal surface. Afterward, each mucosa was continuously rinsed with the 50 mM phosphate buffer pH 6.8 at the flow rate of 1mL/min using a peristaltic pump. A total of 30 mL of phosphate buffer fractions flowing down the mucosa were collected at the time points of 0.5, 1, 1.5, 2, 2.5, and 3 h. In parallel, each 100% reference sample was prepared by rinsing the intestinal mucosa without any adsorbed sample using phosphate buffer and then dissolving each polymer sample in the collected buffer in a concentration of 0.67 mg/mL. All test and reference samples were centrifuged at 13,400 rpm for 5 min at room temperature and fluorescence intensity measurements were taken at an exciting wavelength of 485 nm and an emission wavelength of 535 nm. The percentage mucoadhesion of samples at each time point was calculated by the following equation:

(3) $$\mathrm{Mucoadhesion}\;\left(\%\right)=100-\left(\frac{\mathrm{Fluorescence}\;\left(\mathrm{test}\right)}{\mathrm{Fluorescence}\;\left(\mathrm{reference}\right)}\times 100\right)$$

### 2.11. Mucus Diffusion Study

To investigate the diffusion of the two s-protected thiolated polymers across purified porcine intestinal mucus, the rotating tube method was applied as described previously [27]. In brief, silicon tubes were divided into small pieces of 35 mm length and their one end closed with end caps. Afterward, the silicon tubes were filled with 300 μL of mucus. Fluoresceinamine labeled s-protected thiolated polymers were hydrated in 50 mM phosphate buffer pH 6.8 at a concentration of 1% *m*/*v*. Each solution (20 µL) was added to the open end of the mucus-filled tube and closed with an end cap. After 24 h of incubation time, the tubes were frozen at −80 °C. Afterward, tubes were cut into 3 mm slices and mixed with 200 µL phosphate buffer for 30 min in small tubes and centrifuged for 5 min at 13,400 rpm. The supernatant (100 μL) from each sample was added to 96-well black plates, and fluorescence intensities were measured at an excitation wavelength of 485nm and an emission wavelength of 535 nm with a microplate reader (Tecan infinite^®^ M200 spectrophotometer, Tecan Austria GmbH, Grödig, Austria). Silicon tubes containing mucus without samples served as the negative control. Fluorescence of 20 µL of each test solution mixed with a slice of negative control silicon tube and 200 µL of phosphate buffer served as 100% reference control. The percentage mucus penetration of each sample in various segments was calculated based on the relevant 100% reference fluorescence value.

(4) $$\mathrm{Mucus}\;\mathrm{penetration}\;\left(\%\right)=\frac{\mathrm{Fluorescence}\;\left(\mathrm{test}\right)}{\mathrm{Fluorescence}\;\left(\mathrm{reference}\right)}\times 100$$

### 2.12. Statistical Analysis

All experiments were performed in triplicate. Data were analyzed by GraphPad Prism 5 (Graphpad Software, Inc., San Diego, CA, USA). All data were represented as mean ± SD. Statistical comparisons were made using one-way ANOVA and two-way Bonferroni multiple comparisons test and *p* < 0.05 as the minimal level of significance.

## 3. Results and discussion

### 3.1. Synthesis and Characterization of S-Protected Thiolated PAA Conjugates

**Figure 1:**
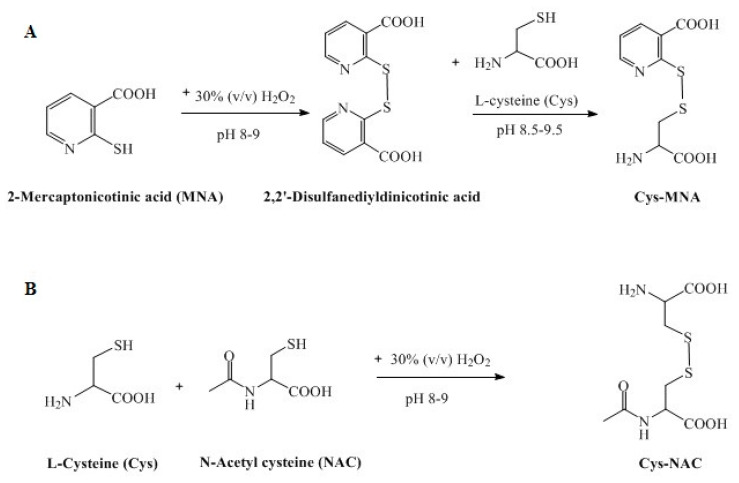
Synthesis of s-protected thiolated ligands: Cys-MNA (**A**) and Cys-NAC (**B**).

This study was based on a comparison of highly reactive and less reactive s-protected thiomers regarding their mucoadhesive properties. PAA with the low molecular mass of 2 kDa was shown to permeate, to a considerable extent, the mucus gel layer and, to some extent, even the underlying tissue. To exclude a systemic uptake of the mucoadhesive polymer, PAA with the medium molecular mass of 450 kDa that can permeate to some extent the mucus gel layer but not the underlying tissue was chosen [25]. As the type of sulfhydryl ligand mainly affects the reactivity of s-protected thiomers, the highly reactive MNA with a thiol pKa 1.87, and the less reactive NAC with a thiol pKa 9.7 were compared within this study. The first step to synthesize s-protected thiolated PAA conjugates was the generation and purification of the s-protected thiol ligands Cys-MNA and Cys-NAC, as shown in Figure 1A,B.

Cys-MNA was prepared and purified using a method that had been established previously [17,18]. The successful formation of Cys-MNA by a disulfide exchange reaction between MNA-dimer and l-cysteine was confirmed by thin-layer chromatography (Figure S1 in Supplementary Materials). The ligand Cys–MNA was the only compound in the reaction mixture that was detected under UV-light and visualized by ninhydrin since it displays a primary amino group.

Cys-NAC ligand was prepared for the very first time and synthesis was confirmed by TLC, ^1^H-NMR, and LC–MS. TLC confirming Cys-NAC formation is shown in Figure S2. In the reaction mixture, Cys-NAC was detected under UV as well as by reaction with ninhydrin. In contrast, cysteine dimer was detected only by reaction with ninhydrin, and NAC dimer was detected just under UV. Neither cysteine nor NAC was detected by TLC in the reaction mixture, confirming their complete consumption in the formation of disulfides. Cysteine dimer was quantitatively removed by filtration as it was completely insoluble over a wide pH range of 2–8. The ^1^H-NMR spectrum of Cys-NAC is shown in Figure S3. Cys-NAC disulfide was characterized by multiplets in the range of 4.56–4.49 and 4.08–4.05 ppm, which are assigned the protons of the CH groups. The multiplets for the methylene protons can be found at 3.40–3.24 and 3.12–2.94 ppm. The methyl protons appear as a singlet at 2.06 ppm.

For further evaluation, the molecular mass of Cys-NAC was determined by LC–MS with positive ESI mode. The mass spectrum as illustrated in Figure S4 showing a major peak at mass to charge ratio (*m*/*z*) of 283.0 representing the protonated form of Cys-NAC that is very close to the theoretically determined molecular mass of non-protonated Cys-NAC disulfide (C_8_H_14_N_2_O_5_S_2_, 282.03).

**Figure 2:**
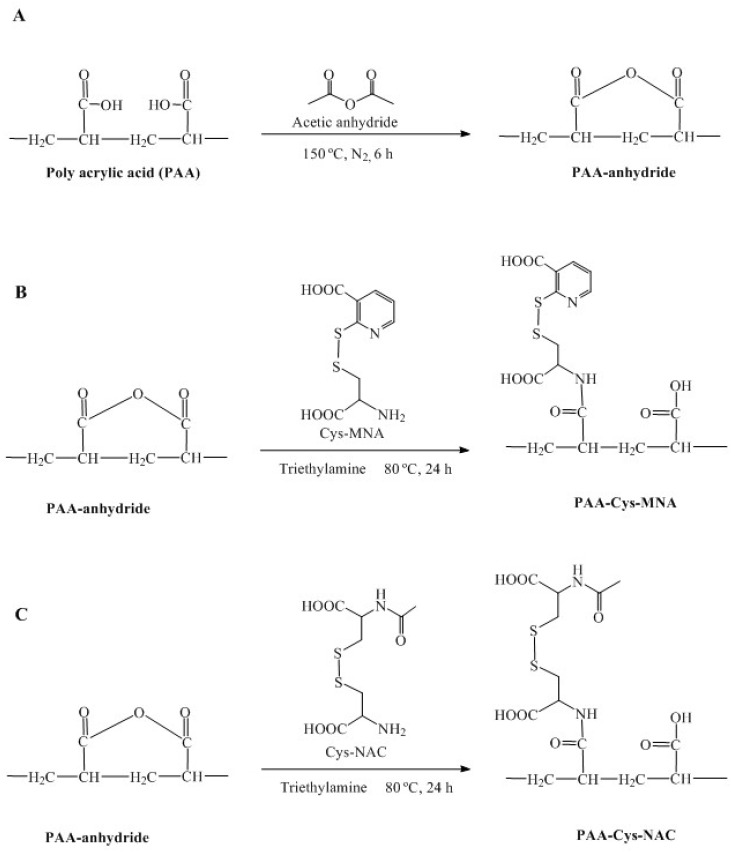
Synthesis of PAA-anhydride (**A**), PAA-Cys-MNA (**B**), PAA-Cys-NAC (**C**).

Both s-protected ligands were covalently attached to PAA via amide bond formation using a novel method. For this purpose, PAA has converted to PAA anhydride as anhydride form is considered highly reactive. In the next step, PAA-anhydride was attached with each thiol ligand without the use of any catalyst. Following dialysis and lyophilization, both thiolated polymers were obtained as light brown fibrous materials. PAA was subjected to the same synthesis process but, without the addition of the respective ligand, served as a control and was obtained as white fibrous material. The chemical synthesis of PAA-anhydride and both thiolated PAA products is illustrated in Figure 2A−C.

**Figure 3:**
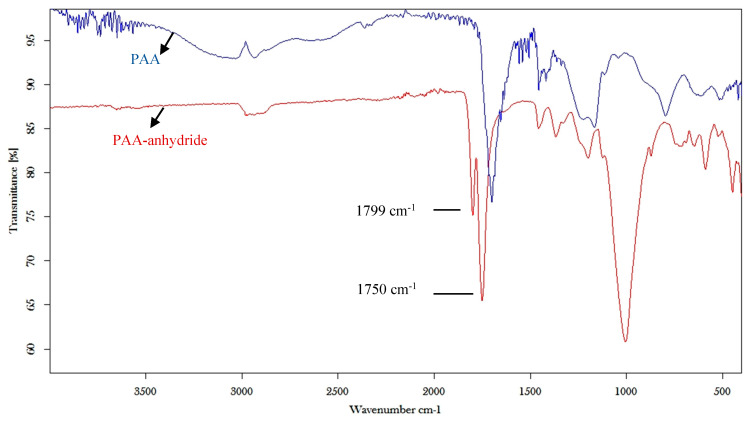
FT-IR spectroscopy of PAA and PAA anhydride.

The structure of PAA anhydride was confirmed by FT-IR spectroscopy by the presence of the two anhydride C=O stretch bands at 1799 and 1750 cm^−1^, as shown in Figure 3.

**Figure 4:**
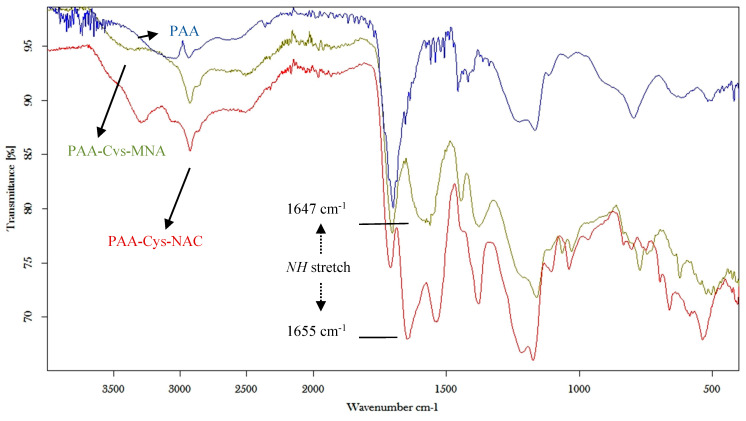
FT-IR spectra of unmodified PAA, PAA-Cys-MNA and PAA-Cys-NAC.

Final products were characterized by FT-IR spectra, as illustrated in Figure 4. The bands at 1647 and 1655 cm^−1^ indicate the NH stretch of the amide bond in PAA-Cys-MNA and PAA-Cys-NAC, respectively.

Furthermore, the amount of free thiols on each s-protected thiolated PAA conjugate, as well as after the reduction of disulfide bonds, was determined. The results are depicted in Table 1. As there was no significant difference in the amount of immobilized thiol groups between the two thiomers, a comparison in their properties being directly related to the chemical structure of each ligand was justified.

### 3.2. Cytotoxicity Studies

**Figure 5:**
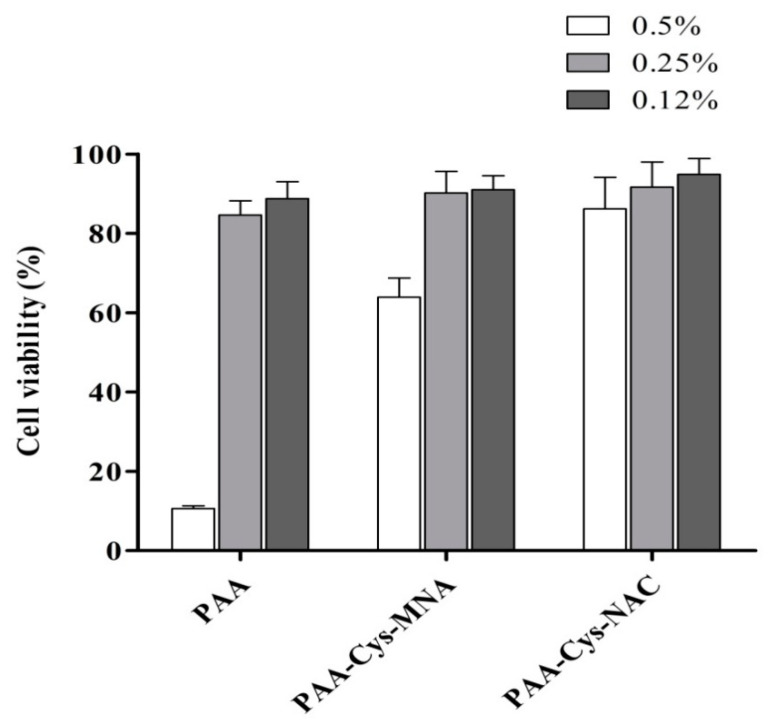
Cell viability of Caco-2 cells after 4 h incubation at 37 °C with PAA and s-protected thiolated PAA conjugates at indicated concentrations using resazurin assay. The data are shown as mean ± SD (n = 3).

In order to evaluate the cytotoxic potential of unmodified and s-protected thiolated PAA conjugates, Caco-2 cells were incubated with three different concentrations of each sample for 4 h. The results of the cytotoxicity studies are illustrated in Figure 5. Generally, cell viability was above 80% for PAA-Cys-NAC at all tested concentrations. In contrast, cell viability of unmodified PAA was <20% at 0.5% *m*/*v* concentration. One possible explanation could be that PAA can form complexes with endogenous Ca^2+^ from intestinal epithelial cells, which might weaken the cellular barrier, leading to cell damage. This complexation depends on the availability of carboxylic groups. In the case of s-protected thiolated PAA, some of the carboxylic groups are not available anymore as they have been transformed to amide bonds with thiol-bearing ligands. As a consequence, their interaction with Ca^2+^ is reduced, resulting in lower toxicity. Unmodified PAA may deprive the cells of Ca^2+^ by forming complexes with extracellular Ca^2+^. This effect might weaken the cellular barrier, leading to cell death [28]. The cell viability of PAA-Cys-MNA was 63% at 0.5% *m*/*v* concentration, whereas, at the same concentration, PAA-Cys-NAC showed 86% cell viability. Comparatively higher toxicity of PAA-Cys-MNA can be attributed to the s-protecting group MNA, whose adverse effects are not fully investigated yet. However, the result confirmed the advantage of using NAC for s-protection, as it is endogenous with a well-established safety profile.

### 3.3. Rheological Investigations 

**Figure 6:**
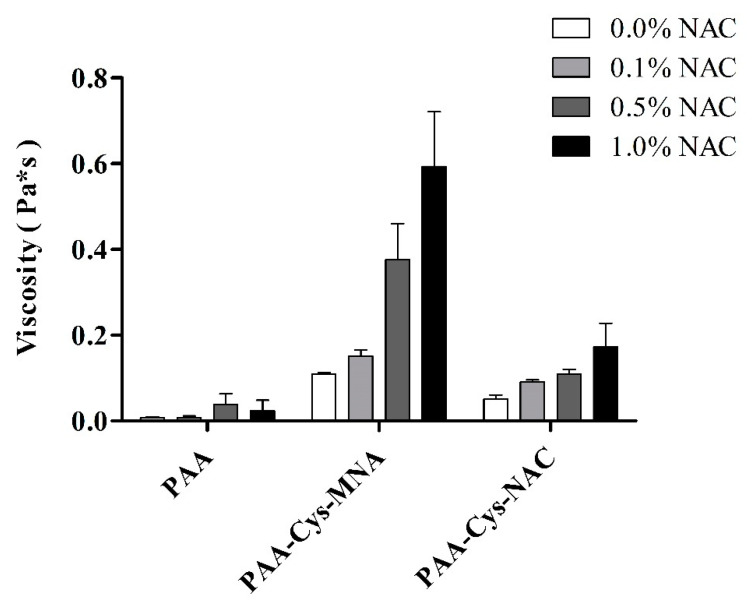
Viscosity of 1% *m*/*v* PAA and s-protected thiolated PAA conjugates after incubation with indicated concentrations of NAC at 37 °C for 1 h. Solutions were prepared in 50 mM phosphate buffer pH 6.8. The data are shown as mean ± SD (n = 3).

Rheological measurements are the common way to evaluate the mucoadhesive strength of thiomers as they reflect the degree of interaction between thiomers and thiol-bearing moieties. The solution of NAC in increasing concentration was used to determine the impact of thiol/disulfide exchange reaction on viscosity improvement. Unmodified PAA and s-protected thiolated PAA conjugates without the addition of NAC were used as respective controls. As shown in Figure 6, an increase in viscosity was negligible in the case of unmodified PAA, whereas PAA-Cys-MNA resulted in an increase in viscosity up to 5.4-fold. In the case of PAA-Cys-NAC, viscosity was increased up to 3.4-fold. As the increase in viscosity is based on thiol/disulfide exchange reactions within the thiomers triggered by the thiol group of NAC, the higher extent of viscosity increase in the case of PAA-Cys-MNA can be explained by the higher reactivity of its s-protected thiol ligands in comparison to PAA-Cys-NAC.

**Figure 7:**
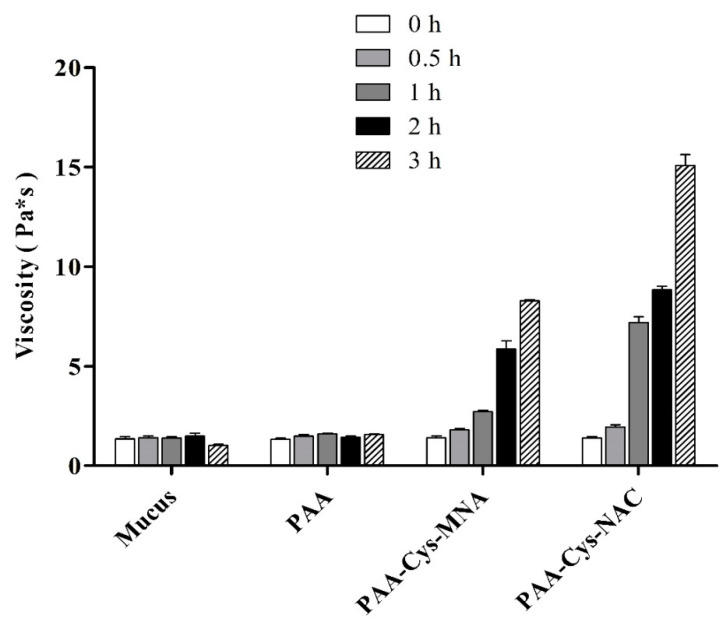
Viscosity of 1% *m*/*v* PAA and s-protected thiolated PAA conjugates incubated with mucus at 37 °C for indicated time points. Solutions were prepared in 50 mM phosphate buffer pH 6.8. Mucus incubated with buffer was used as control. The data are shown as mean ± SD (n = 3).

**Figure 8:**
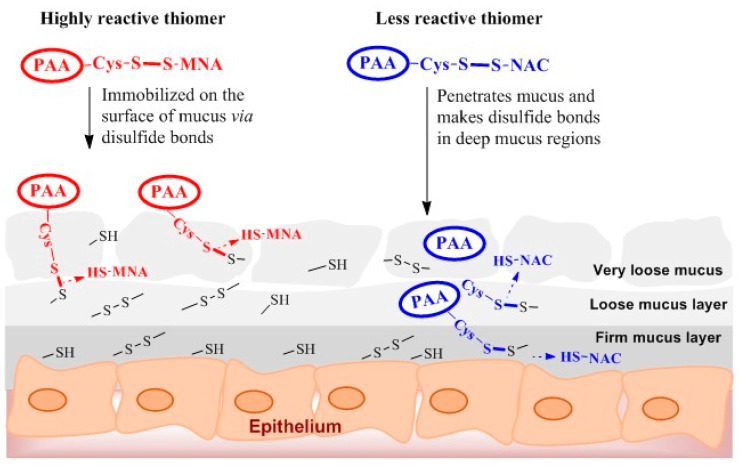
Schematic illustration of thiol/disulfide exchange reactions of highly reactive PAA-Cys-MNA and low reactive PAA-Cys-NAC with thiol moieties in mucus layer.

The viscosity of mucus was also measured after the addition of PAA and s-protected thiolated PAA conjugates at different time points as shown in Figure 7. As illustrated, in comparison to PAA, s-protected thiolated PAA conjugates effectively enhanced the viscosity of mucus within 3 h. Mucus viscosity was statistically (*p* < 0.05) higher (10.9-fold) with PAA-Cys-NAC as compared with PAA-Cys-MNA (5.6-fold). We can conclude that PAA-Cys-MNA strongly interacts with superficial thiol groups of mucus, hindering further mucus penetration, whereas PAA-Cys-NAC can deeply interpenetrate, resulting in more intensive interactions with mucus. It was observed that the viscosity of PAA-Cys-NAC was more efficiently enhanced in the presence of mucus than NAC. This difference can be explained by the higher extent of crosslinking due to disulfide exchange reactions taking place between cysteine-rich subdomains of mucins and deeply penetrated PAA-Cys-NAC. In contrast, in the presence of NAC exhibiting just one thiol group, disulfide/thiol exchange reactions do not occur so rapidly and efficiently. The mechanism of thiol/disulfide exchange reactions taking place within the mucus has been schematically represented in Figure 8. As shown, the highly reactive thiomer is strongly entrapped in the loose mucus layer due to rapid interactions and disulfide bond formation with thiol moieties. The less reactive PAA-Cys-NAC can likely interpenetrate the mucus gel layer to a higher extent before getting immobilized via thiol/disulfide exchange reactions in the firm mucus layer. The lower reactivity and deeper interpenetration results in a higher mucoadhesion of less reactive thiomers. The validity of this mechanism has also been confirmed by Netsomboon et al., who showed higher mucoadhesion for a less reactive s-protected chitosan than the corresponding highly reactive thiomer [29].

### 3.4. Swelling Behavior

**Figure 9:**
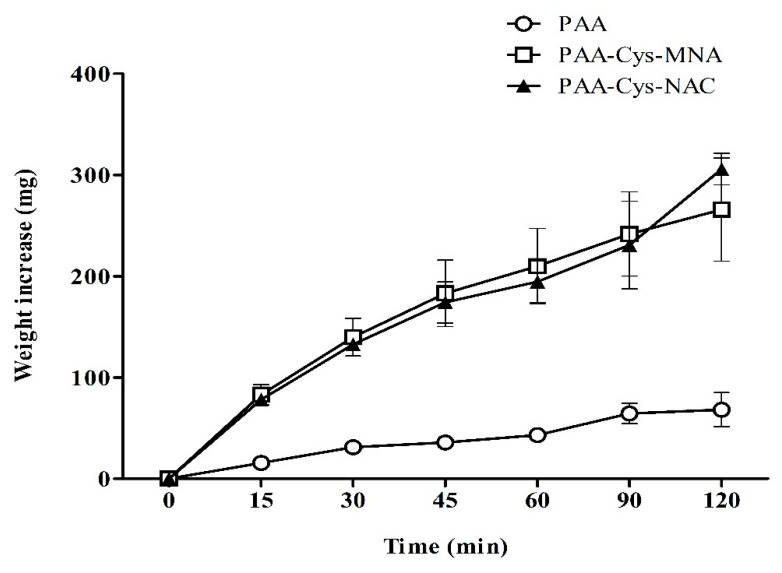
Swelling behavior of unmodified PAA and s-protected thiolated PAA conjugates. The study was carried out in 50 mM phosphate buffer pH 6.8. The data are shown as mean ± SD (n = 3).

Mucoadhesive polymers are well known to absorb water from the underlying mucosal layer. As this process also has a substantial impact on mucoadhesion properties, water uptake studies were carried out with disks of unmodified PAA, PAA-Cys-MNA, and PAA-Cys-NAC. The results, as illustrated in Figure 9, provide evidence that the attachment of thiol ligands strongly influences the water uptake and swelling behavior of PAA. Unmodified PAA showed significantly less (*p* < 0.05) swelling than both s-protected PAA conjugates. Both s-protected thiomers showed a constant increase in the water uptake for 2 h. A high water uptake favors the inter-diffusion process between the mucus layer and polymer resulting in stronger mucoadhesion [30]. It also denotes less crosslinking within the thiomer itself, favoring interactions between the thiomer and the mucus layer [24]. 

### 3.5. In-Vitro Mucoadhesion Studies

**Figure 10:**
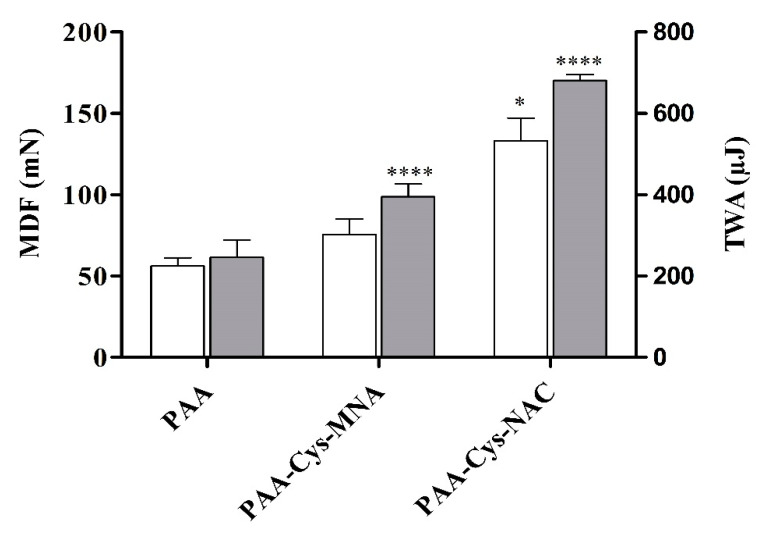
Tensile studies on porcine small intestinal mucosa. The MDF and TWA of the test disks of unmodified PAA and s-protected thiolated PAA conjugates on intestinal mucosa were measured with a texture analyzer. White bars depict MDF and light gray bars represent TWA. The data are shown as mean ± SD (n = 3).

Tensile studies were performed to evaluate the adhesive features of unmodified and s-protected thiolated PAA conjugates on intestinal mucosa as illustrated in Figure 10. PAA-Cys-MNA resulted in a significant 1.4-fold improved MDF and 1.6-fold improved TWA as compared to unmodified PAA. Similarly, PAA-Cys-NAC resulted in the significantly higher (*p* < 0.05) MDF (2.4-fold) and TWA (2.8-folds) as compared to unmodified PAA.

**Figure 11:**
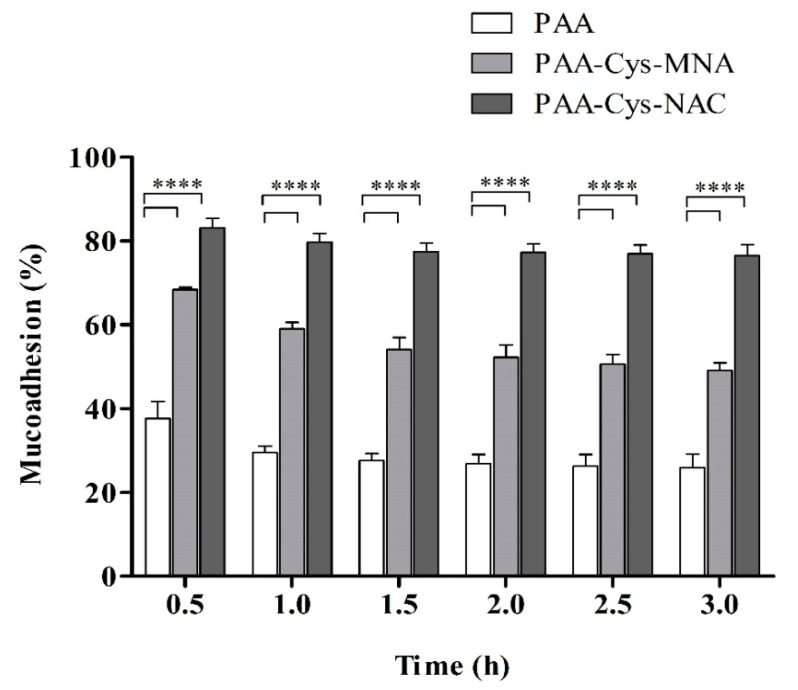
Percentage of remaining unmodified PAA and s-protected thiolated PAA on porcine intestinal mucosa continuously rinsed with 50 mM phosphate buffer pH 6.8 at 37 °C and 100% relative humidity at indicated time points. The data are shown as mean ± SD (n = 3).

Mucoadhesive properties were further evaluated by the flow-through method. The basic principle of this mucoadhesion test is the quantification of PAA conjugates detaching from the mucosa as a function of time [31]. The results of the mucoadhesive studies on porcine intestinal mucosa, as illustrated in Figure 11, showed higher mucoadhesion of s-protected thiolated PAA than unmodified PAA. These mucoadhesive properties are in accordance with previous findings, which strongly support the notion that a higher degree of thiolation improves mucoadhesion [16,26]. The results confirm that about 76.5% of PAA-Cys-NAC and 49% of PAA-Cys-MNA conjugates remain attached to the intestinal mucosal surface after 3 h, whereas unmodified PAA remains attached up to 25.8%. It can be shown from these results that the mucoadhesive properties of PAA-Cys-NAC were 1.6- and 3-fold improved as compared to PAA-Cys-MNA and unmodified PAA, respectively. 

Thiolated polymers get attached to the mucosa mainly via disulfide exchange reactions with cysteine-rich domains of the mucus layer. In addition to these disulfide exchange reactions, some weak electrostatic or van der Waals interactions may also be involved in mucoadhesion [7]. As both s-protected thiomers showed similar swelling behavior, as shown in Figure 9, the comparatively higher mucoadhesive properties of PAA-Cys-NAC can only be attributed to its more interpenetration within mucus. The higher degree of interpenetration provides a greater surface area for non-covalent and covalent interactions, resulting in a stronger adhesion of the comparatively less reactive s-protected thiomer. Once in contact, PAA-Cys-MNA rapidly interacts with mucus based on disulfide exchange reactions that hinder their deep penetration into mucus, whereas the reactivity of PAA-Cys-NAC is not too high to hinder its deep interpenetration. Due to this deep interpenetration, it can also interact with the firm mucus layer adhering to the epithelial layer, which prevents its easy washout from the mucosal surface. Moreover, the protection of thiol groups in s-protected thiolated PAA conjugates does not only prevent early oxidation of thiols but also enhances mucosal contact time, whereby more active thiol groups are available for longer periods of intimate contact with the mucosal membrane in many different ways [9]. Thiolated polymers have already shown great potential in various clinical trials [16] and several products containing thiolated polymers have already successfully entered the global health care market. Their potential might even be further improved by the attachment of less reactive thiol ligands to the polymer backbone, as described in this study. Moreover, taking into consideration that the released substructure NAC is generally regarded as safe underlines the clinical relevance of our findings. 

### 3.6. Mucus Diffusion

**Figure 12:**
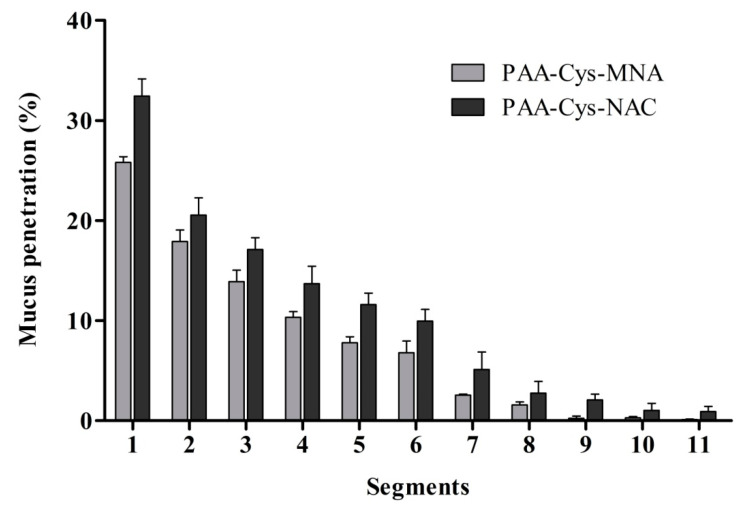
Mucus diffusion studies of fluoresceinamine-labeled s-protected thiolated PAA conjugates using porcine intestinal mucus by the silicon tube method. The data are shown as mean ± SD (n = 3).

s-protected thiolated PAA conjugates were applied to mucus-containing silicon tubes as the elongated mucus barrier model in order to investigate the diffusion ability of these compounds into deeper regions of the mucus gel layer. The results of this study are illustrated in Figure 12. They were in good agreement with previous findings regarding the mucus diffusion behavior of thiolated polymers [32,33]. In all segments, diffusion was significantly higher for PAA-Cys-NAC than that of PAA-Cys-MNA, confirming the benefit of less reactive thiol ligands for the mucus interpenetration of thiomers. These results are also in good agreement with water uptake studies illustrated in Figure 9 and the mucoadhesion shown in Figure 11. As described previously, PAA-Cys-MNA rapidly interacts with mucus on the surface, which hinders its further diffusion into deeper mucus regions, whereas PAA-Cys-NAC shows higher mucus diffusion due to less reactivity.

## 4. Conclusions

Mucoadhesion is a complex process that involves in the case of thiomers, non-covalent and covalent interactions [22]. In this regard, the interpenetration of polymeric chains is of particular importance, since a higher degree of interpenetration will certainly lead to increased non-covalent/covalent interactions of polymers with mucus resulting in higher mucoadhesion. Theoretically, the medium to low reactivity of thiomers can be advantageous to facilitate mucus interpenetration [16]. In this study, we synthesized a less reactive s-protected thiomer, PAA-Cys-NAC, and provided a proof-of-concept that it is a greater mucoadhesive than the well-established, highly reactive s-protected thiomer PAA-Cys-MNA. The mucoadhesion of PAA-Cys-NAC was 3-fold higher on intestinal mucosa in comparison to unmodified PAA and about 1.6-fold higher than PAA-Cys-MNA. According to these results, the Cys-NAC ligand seems to be a promising tool for the design of less reactive but highly mucoadhesive s-protected thiomers.

## Figures and Tables

**Table 1:** Amount of free thiols and thiols after reduction with NaBH_4_ for s-protected thiolated PAA conjugates. The data are shown as mean ± SD (n = 3).

Product	Free Thiols (µmol/g)	Thiols after Reduction with NaBH_4_ (µmol/g)
PAA-Cys-MNA	43.22 ± 21	660.65 ± 42.7
PAA-Cys-NAC	20.84 ± 15	683.50 ± 27.4

## Supplementary Materials

The following are available online at https://www.mdpi.com/2073-4360/12/6/1259/s1, Figure S1: TLC of Cys-MNA disulfide; (**A**) under UV, (**B**) ninhydrin spray; (1) Cys, (2) MNA and (3) reaction mixture, Figure S2: TLC of Cys-NAC disulfide; (**A**) under UV, (**B**) ninhydrin spray; (1) Cys, (2) Cys dimer, (3) NAC, (4) NAC dimer and (5) reaction mixture, Figure S3: ^1^H-NMR spectrum of Cys-NAC, Figure S4: Mass spectrum of Cys-NAC disulfide determined by LC-MS with positive ESI mode.
