# Supplementary material for: Less Reactive Thiol Ligands: Key towards Highly Mucoadhesive Drug Delivery Systems
Source: Polymers (Basel). 2020 May 30;12(6):1259. doi: 10.3390/polym12061259 (PMC7362194; doi:10.3390/polym12061259)
Supplement: Supplementary file 1 [file polymers-12-01259-s001.pdf]

Supplementary Data

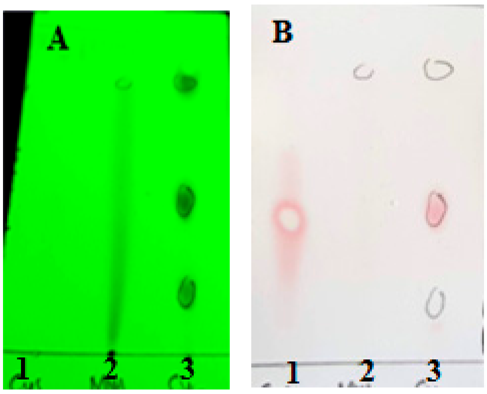

Figure S1. TLC of Cys-MNA disulfide; (**A**) under UV, (**B**) ninhydrin spray; (1) Cys, (2) MNA and (3) reaction mixture.

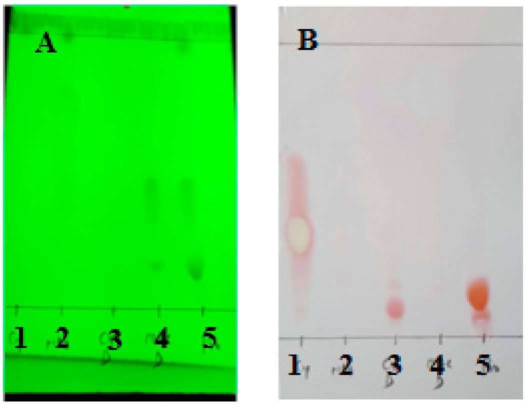

Figure S2. TLC of Cys-NAC disulfide; (**A**) under UV, (**B**) ninhydrin spray; (1) Cys, (2) Cys dimer, (3) NAC, (4) NAC dimer and (5) reaction mixture.

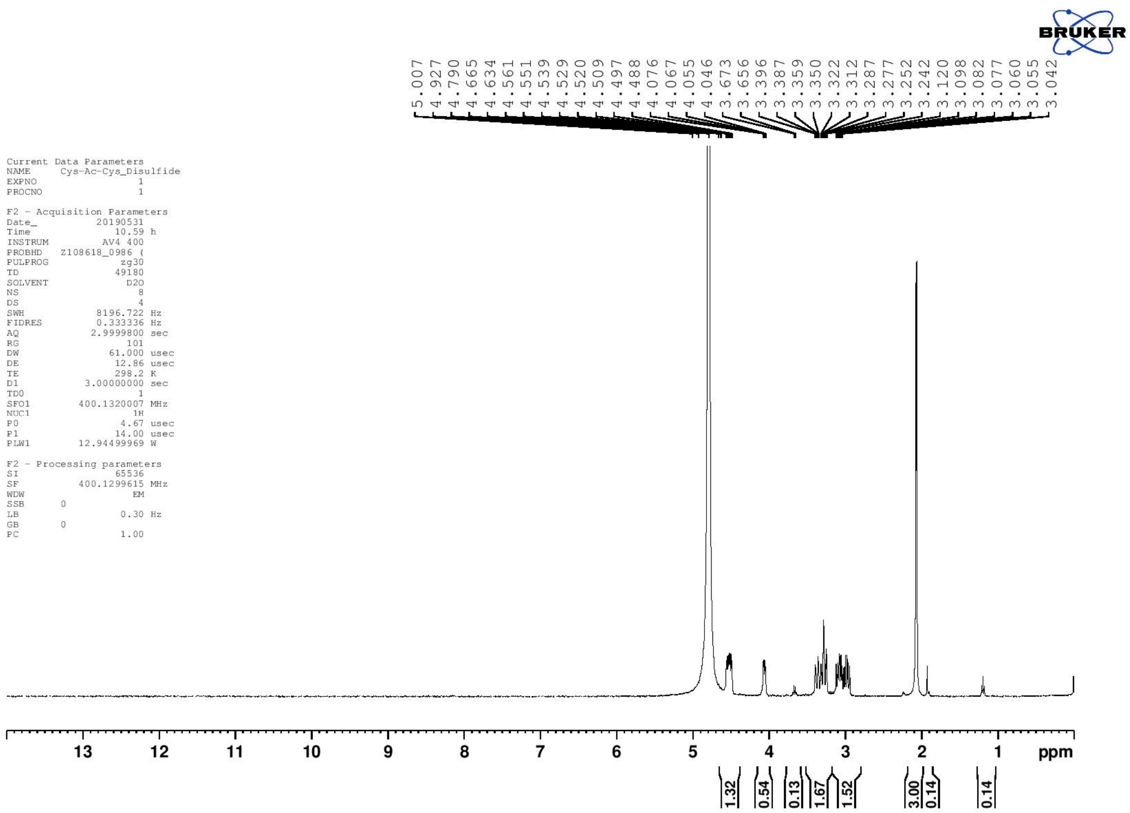

Figure S3. ^{1}H-NMR spectrum of Cys-NAC

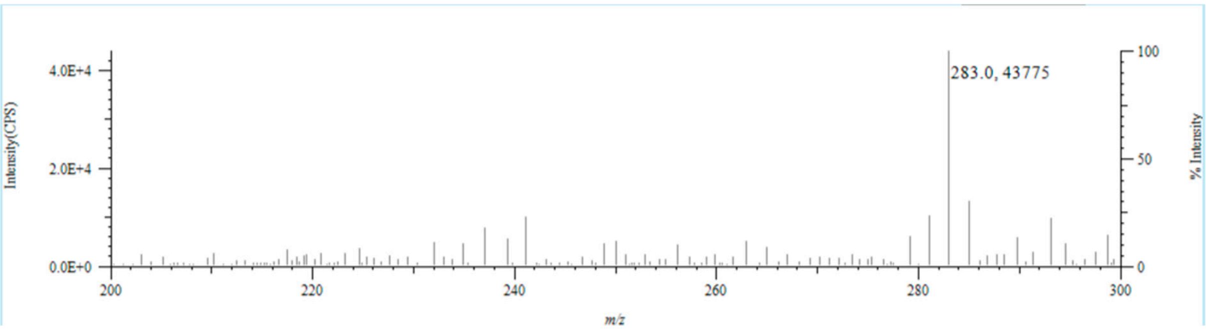

Figure S4. Mass spectrum of Cys-NAC disulfide determined by LC-MS with positive ESI mode
